# Role of Manganese Oxyhydroxides in the Transport of Rare Earth Elements Along a Groundwater Flow Path

**DOI:** 10.3390/ijerph16132263

**Published:** 2019-06-26

**Authors:** Haiyan Liu, Huaming Guo, Olivier Pourret, Yi Chen, Rongxiao Yuan

**Affiliations:** 1 School of Water Resources and Environmental Engineering, East China University of Technology, Nanchang 330013, China; 2 State Key Laboratory of Biogeology and Environmental Geology, China University of Geosciences, Beijing 100083, China; 3 UniLaSalle, AGHYLE, 60026 Beauvais Cedex, France

**Keywords:** Lanthanides, China, Sorption, Modeling, Aquifer, Cerium anomaly, Critical zone

## Abstract

Rare earth elements (REE) are known to be emerging contaminants in hydrosphere, but roles of hydrous manganese oxyhydroxides (HMO) in REE transport in groundwater remains unknown. In this study, groundwater was sampled along a flow path in the North China Plain to determine the behavior of REE surface complexation to HMO by a modeling and field study approach. Results show that the proportion of neodymium (Nd) complexed by HMO ranges from 0.2% to 95.8%, and from 0.3% to 99.6% in shallow groundwater and deep groundwater, respectively. The amount of complexed REE increases along the flow path. REE bound to HMO exhibit decreasing trends with increasing atomic number. The process was determined to be independent of pH, HMO content, and metal loading. This finding further demonstrates HMO-REE complexation plays a key role in transport of REE in groundwater through preferential scavenging of light REE (LREE) over heavy REE (HREE). Nevertheless, carbonate ligands appear to be robust competitors in reducing the amount of REE sorbed to HMO when solution pH rises above 8.0. Assuming that 50% of Mn concentration occurs as HMO, the amount of complexed REE was predicted to show a more marked decrease in LREE compared to that of HREE.

## 1. Introduction

The rapid development of modern technologies has been accompanied by an increase of rare earth elements (REEs) released into the environment. Natural water containing REE (i.e., Gd) at concentrations far above their background values occur worldwide, including the USA [1,2], Australia [3], England [4], Brazil [5], Germany [6], France [7], Italy [8], the Czech Republic [9], Japan [10], and Korea [11]. In China, REE have been considered one of the main chemical contaminants since the 1990s [12]. In the past decades, the anomalously high REE concentrations being commonly detected in water resources has raised global concern due to their potential for long-distance transport, toxicity, and bioaccumulation [13,14]. This issue has been accentuated by a steep increase in REE demand which leads to a growing environmental discharge of REE, posing a potential risk to human health and ecological system [15,16]. Therefore, full understanding of the fate and transport of REE in hydrosystems is an important issue to protect human health and support sound environmental policy-making.

Research in REE abundance and fractionation in groundwater has been conducted over the last three decades [17,18,19,20,21,22,23]. It is commonly recognized that the mobilization and transport of REE is impacted by reactive solids via strong liquid–solid phase interactions. Processes such as REE co-precipitation with secondary minerals [24,25], REE adsorption onto ferromanganese oxides [26], and amorphous ferromanganese precipitation [27] sequestrate, to a large extent, the aqueous REE from bulk solution. This results in low REE concentrations (e.g., from 10^−9^ to 10^−14^ mol/L) in near neutral groundwater. Indeed, to investigate the REE interaction with Fe precipitates, experiments on REE scavenging were conducted by Bau [28], showing that the scavenged REE increased from approximately 10% at pH < 4.6 to more than 90% at pH > 6 in the presence of dissolved Fe oxyhydroxide. This result is in contrast with the behavior of REE in the absence of Fe-oxyhydroxides, where the scavenged REE were below 4.6%. Dissolved carbonate ion (CO_3_^2−^) has been shown to influence the solution complexation and partition coefficients describing the sorption of REE on amorphous ferric hydroxide [29,30,31]. The enhanced REE solution complexation by carbonate ions was reported to be a mechanism of the Fe distribution coefficient decrease [31].

In-depth understanding of geochemical behavior of REE requires more comprehensive and quantitative information on the REE surface and solution complexation mechanisms. Numerous studies have been devoted to understanding REE surface complexation with Fe oxides [28,30,32,33,34,35], while less effort has been made toward the quantitative study of REE surface complexation with Mn oxides; the limited existing information suggested that REE binding with Mn oxides could be delineated using a two-site model [36]. The proposed model was recently applied to a field study on the fractionation and mobility of REE in groundwater. The results indicated that REE showed a greater affinity for Mn oxides as compared to Fe oxides [23]. Complexation with both of these oxides, however, could be a net sink of aqueous REE [37].

Early laboratory experiments dedicated to the mechanism of REE incorporation into natural Mn oxides were carried out by Koeppenkastrop and De Carlo [38]. The study indicated that the REE sorptive behavior showed a preferential uptake by Mn oxides of light REE (LREE) relative to heavy REE (HREE) and suggested that this fractionation trend resulted in an LREE enrichment in the solid phase. The study by Koeppenkastrop and De Carlo [38] further described a significant decoupling of Ce from neighbors La and Pr, which was not observed on Fe oxide surfaces (i.e., goethite). Based on these observations, the effect of dissolved carbonate ion complexation on the sorption kinetics of REE [39] as well as their fractionation quantified by Sm/Yb ratio was evaluated [40]. A greater extent of REE fractionation was initially observed on Mn oxides (i.e., δ-MnO_2_) compared to that on Fe oxides (i.e., FeOOH) in the presence of dissolved carbonate ligands [39]. More recently, REE complexation with hydrous manganese oxyhydroxides (HMO) was quantitatively determined using the diffuse double-layer sorption model [36]. The results obtained from the modeling study showed that LREE bound onto HMO increased with increasing pH and ionic strength and a fractional REE pattern exhibited large positive Ce anomalies and a downward declining convex tetrad effect [27,41,42].

Little research has been published on REE speciation and transport along groundwater flow paths considering REE complexation to HMO, in spite of the numerous studies concerning the behavior of REE along groundwater flow paths/transects [20,21,25,43,44,45,46,47]. The only studies quantifying REE binding to HMO were that of Decrée et al. [48] and Liu et al. [23]. The former authors calculated REE speciation in a metal-ligand groundwater system over a range of pH from 4 to 12 by considering HMO concentrations of 10^−4^ mol/L and 10^−5^ mol/L, respectively, while the latter study compared the impact of HMO to HFO on REE speciation in groundwater along a flow path. Frequently, Mn has been used as a redox sensitive component for qualitative evaluation of processes controlling REE mobilization [43,47]. However, it is essential to quantitatively describe REE binding to Mn-oxyhydroxides, in order to better understand the fate and transport of the REE in groundwater systems and to determine the extent to which REE groundwater patterns depend on fractionation by HMO. Therefore, the objectives of this study are to (i) identify the roles of HMO in transport of REE in groundwater along the flow path; (ii) quantitatively determine REE surface complexation reactions with HMO in groundwater from different aquifers; and (iii) evaluate the influence of HMO concentrations on REE speciation and fractionation.

## 2. Materials and Methods

### 2.1. Regional Hydrogeological Settings

**Figure 1:**
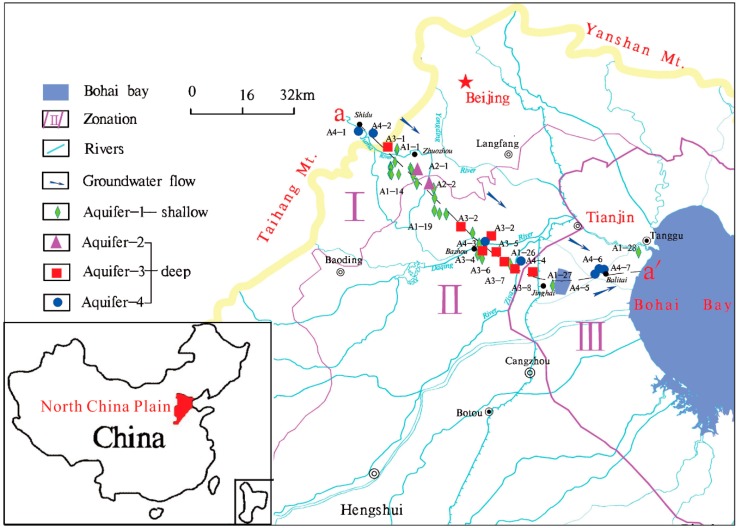
Study area and sampling locations (I, II, and III correspond to the piedmont plain, the central plain, and the littoral plain, respectively).

The North China Plain (NCP) is within the longitudes of 112°30’ to 119°30’ E and the latitudes of 34°46’ to 40°25’ N and has a total area of approximately 32 × 104 km^2^. The NCP is bounded by the Yanshan Mountain to the north, by Taihang to the west, by the Yellow River to the south, and by Bohai Bay to the east (Figure 1). The NCP is a semi-arid region with a middle-latitude continental monsoon climate. The annual average temperature is from 12 °C to 13 °C, and annual precipitation ranges from 400 to 1200 mm, with a mean value of approximately 550 mm [49]. The mean annual potential evaporation is 1645 mm [50].

Geographically, the plain generally inclines eastward from an altitude of approximately 100 m above land surface (a.l.s) in the west to approximately 1 m a.l.s in the east with underlying sedimentary rocks ranging in age from Archaean to Cenozoic. Three hydrogeological units from the piedmont zone to the littoral area correspond to piedmont alluvial–proluvial plain, central alluvial–lacustrine plain, and eastern alluvial–littoral plain [51] (Figure 1). The Cenozoic sediments are dominated by medium-coarse sand and gravel deposits in the piedmont area. The alluvial–proluvial fans occur as a ribbon along the bases of the Taihang and Yanshan mountains. The central plain mainly consists of fine-grained, silty and clayey deposits. The littoral plain is predominated by alluvial deposits sandwiched with locally abundant marine deposits along the western and northern coast of Bohai Bay. According to the investigation of Chen and Ni [52], aquifer minerals are dominated by quartz, K-feldspar, and hornblende in the piedmont, and calcite accounts for 5%–14%. Clay minerals such as kaolinite, illite, and montmorillonite are more common in the central and littoral plain than in the piedmont plain [51].

**Figure 2:**
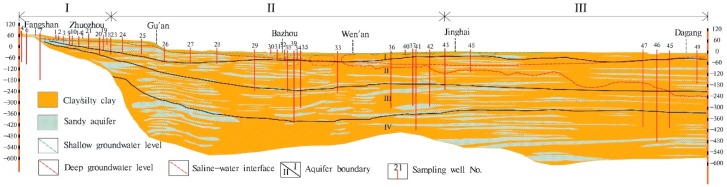
Schematic hydrogeological cross-section of the sampled path. Investigated wells and their depths are indicated by vertical numbered lines (e.g., 21 represents No. 16-21). Boundaries of the aquifers I~IV at different depths are shown using black solid lines.

Regional Cenozoic groundwater occurs in four aquifers composed of sandy gravel, medium-fine, and fine sand. According to their lithology, hydrodynamics and geologic age, the four aquifers include the Holocene formation (aquifer-I), the late Pleistocene formation (aquifer-II), the middle Pleistocene formation (aquifer-III), and the early Pleistocene formation (aquifer-IV) from top to bottom [53] (Figure 2). The depth of these four aquifers is shown in the hydrogeological cross section (Figure 2). The aquifers in the piedmont have a thickness of about 150 m with fluvial deposits being dominated, whereas in the central and littoral plains, the aquifer sediments are mainly composed of alluvial and lacustrine deposits with a thickness up to 600 m. In the piedmont plain, the aquifers (Aquifer-I~IV) are connected and mainly unconfined, establishing a local flow system. In the latter two plains, Aquifers-II, -III, and -IV (deep aquifers) are confined, combined to Aquifers-I (shallow aquifer) forming a regional flow system.

Groundwater system is recharged by precipitation, irrigation return flow, lateral inflow from the Taihang Mountains, and infiltration of surface water flow [54]. Shallow groundwaters within the Taihang Mountains discharge into rivers and canals during the course of downward migration. Deep groundwaters constitute the drinking water supply and agriculture irrigation, and thus their flows are greatly impacted by anthropogenic activity (e.g., pumping), especially since 1980, when deep groundwaters were diverted in cities and agricultural areas due to the depletion of shallow groundwaters and surface waters. Irrigation return flow can be an important component substantially contributing to the recharge in the center and littoral plains [54], and artificial exploitation is a major discharge pathway for deep groundwater.

### 2.2. Groundwater Sample Collection

Forty-six groundwater samples were collected from electric-powered public wells used for drinking water supply or agricultural irrigation with 29 samples from shallow aquifers and 17 from deep aquifers. These samples were taken approximately along the profile a-a’ from Shidu, through Zhuozhou and Bazhou, to southern Tianjin, over a total distance of approximately 188 km (Figure 1). The depth of the investigated wells spans from 4.5 m to 500 m b.l.s (Figure 2). Before sampling, all wells were pumped for at least 20 min (irrigation wells for several hours due to ongoing agricultural irrigation) until water pH, temperature, electrical conductivity (EC), and oxidation–reduction potential (ORP) remained stable. Groundwater was stored in sample vessels which were all pre-cleaned using acid-washing and deionized water in the laboratory and rinsed three times using extracted groundwater. The 0.45-μm-membrane filters were used for water filtration in the field. The filtered groundwaters, used for analysis of major cations (Ca^2+^, Mg^2+^, K^+^, Na^+^), REE and other trace elements, were stored in 100 mL of high-density polyethylene bottles and immediately acidified to a pH < 2.0 by addition of 6 mol/L of purified-HNO_3_. Groundwaters used for major anion analysis were sampled without acidification. Groundwater samples for dissolved organic carbon (DOC) analysis were collected in 30-mL amber glass bottles and immediately acidified with 1:9 (volume) H_2_SO_4_ to pH < 2.0. All samples were stored at 4 °C prior to analysis.

### 2.3. Groundwater Sample Analysis

A portable multi-meter (HANNA, HI 9828) was used to determine physico-chemical parameters, including T, pH, EC and ORP in the field. The instrument was calibrated using standard solutions before use. By letting water flow through an in-line flow cell with minimal atmospheric contact, groundwater physico-chemical conditions were maintained during field monitoring. Alkalinity was titrated on-site using a Model 16900 digital titrator (HACH) with standard purified H_2_SO_4_ (0.80 mol/L) and bromocresol green-methyl red indicator. The results obtained were compared to a previous study [55] for validation. A portable UV/VIS spectrophotometer (HACH, DR2800) was used to determine concentrations of total Fe, Fe(II), nitrite, ammonium, and sulfide in the field.

Groundwater major anion (e.g., Cl^−^, NO_3_^−^, and SO_4_^2−^) concentrations were determined by using an ion chromatography system (ICS2000, Dionex, Thermo Fisher Scientific, Waltham, USA), with a precision better than 3%. For major cation and trace element analysis, ICP-AES (iCAP6300, Thermo Fisher Scientific, Waltham, USA) and ICP-MS (7500C, Agilent Technologies, Santa Clara, USA) were used, respectively. Samples with elevated Mn concentrations were diluted appropriately to fit the standard curve during analysis. Validation of chemical data using the charge balance method showed that all tested samples had a precision better than 5%.

Groundwater REE concentrations were quantified using ICP-MS (7500C, Agilent Technologies, Santa Clara, USA) following the routine protocol described by Guo et al. [45] and Liu et al. [47]. Briefly, seven REE standard solutions (1 ng/L, 2 ng/L, 10 ng/L, 100 ng/L, 250 ng/L, 500 ng/L, and 1000 ng/L), diluted from a stock standard (10 mg/L), were used to calibrate the ICP-MS. A duplicate standard sample was analyzed every 10 samples to check the analytical quality of the determination during analysis. In addition, two REE standard reference water samples (PPREE1 and SCREE1) were routinely run to verify the accuracy of the analytical method. The analytic precision for the standard reference solutions was better than 3% relative standard deviation and for sample REE analyses was generally better than 5%.

### 2.4. Speciation Modeling

The speciation modeling was performed with hydrogeochemical code PHREEQC version 3.3.9 [56] using the Nagra/PSI database [57] according to the method described previously [36]. The PHREEQC database was updated by the incorporation of well-accepted stability constants at 0 ionic strength at 25 °C for REE inorganic anion complexation. The values of the constants have been outlined in Liu et al. [58]. REE complexation to HMO was taken from Pourret and Davranche [36]. Detailed information on the processing technique has been described in Pourret and Davranche [36]. Briefly, HMO were obtained via precipitation equilibrium with the Mn content determined in groundwater collected from different zones. Both strong and weak binding sites formed upon the precipitation of HMO. Thus, REE-HMO surface complexation modeling was performed in the presence of Mn oxyhydroxides (i.e., pyrolusite 95%/manganite 5% as evidenced by X-ray diffraction, data not shown) by holding the physiochemical parameters (e.g., pH, Eh, and major ions). For the surface complexation model (SCM), surface-complexed and diffuse layer species were taken as the components of the system, in which groundwater flow was not taken into account. Manganese carbonate was not considered in the modeling as the pH of the samples was generally lower than 8.5 and even if present it does not affect the saturation index of Mn oxyhydroxides [59]. All REEs considered in the model were trivalent, because oxidation of Ce(III) occurs after initial sorption onto mineral surface sites [28].

## 3. Results

### 3.1. Groundwater General Chemistry

**Figure 3:**
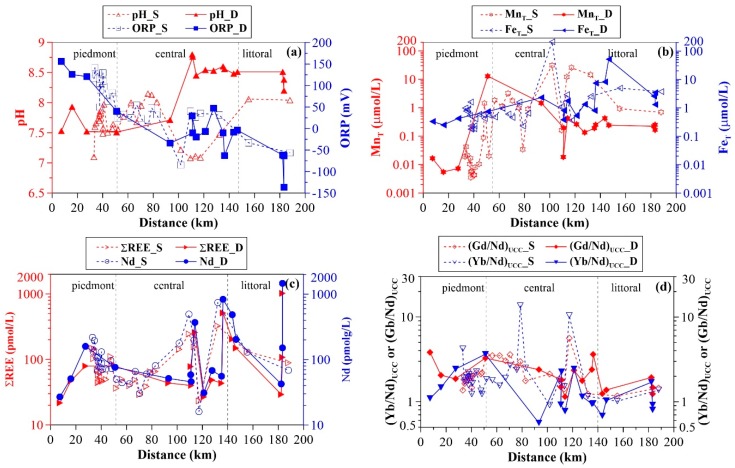
Groundwater parameters as a function of distance from the recharge area ((**a**): pH and ORP; (**b**): total Mn (Mn_T_) and total Fe (Fe_T_); (**c**): total REE (∑REE) and Nd; and (**d**): (Gd/Nd)_UCC_ and (Yb/Nd)_UCC_).

The physicochemical parameters of all groundwater samples are presented in the Supplementary Materials (Tables S1 and S2). Groundwater samples are neutral to weakly alkaline, with pH values varying from 7.1 to 8.2 in shallow groundwater, and from 7.5 to 8.8 in deep groundwater. In general, the pH increases from the piedmont to the littoral plain (Figure 3a), except for shallow groundwater samples in the central plain (100 to 120 km) that had low-pH anomalies, which may be the result of mixing of newly recharged waters (i.e., acidic rain water). Average bicarbonate concentrations of 6.93 mmol/L and 5.00 mmol/L were recorded for the shallow and deep groundwater samples, respectively. Along the flow path, shallow groundwater HCO_3_^−^ concentrations show an overall increasing trend (Table S1), while no substantial variation is observed for deep groundwater samples. However, lower concentrations of 0.52 mmol/L are found in the central plain (Table S2). Chloride concentrations generally increase along the groundwater flow path, whereas sulfate varies substantially within the range of from 0.15 mmol/L to 30.08 mmol/L, and 0.12 mmol/L to 2.77 mmol/L for shallow and deep groundwaters, respectively. Elevated SO_4_^2–^ concentrations in groundwater samples, greater than 2.60 mmol/L, indicated by the Chinese drinking water guideline, occur in the central plain from 80 to 120 km from the recharge zone. Groundwater cations are dominated by Ca^2+^and Mg^2+^ in the piedmont plain. However, Na^+^ becomes predominant with the down-gradient flow along the flow path for both shallow and deep groundwater samples. Consequently, the groundwater hydrochemical phases evolve from HCO_3_-Ca-Mg in the recharge zone to SO_4_-Cl-Na or Cl-Na in the discharge zone.

The oxidation–reduction potential (ORP) ranges from –85 mV to 142 mV (average 56 mV) and from –136 mV to 156 mV (average 6 mV) in shallow and deep groundwaters, respectively. Variation of ORP is quite similar between the shallow and deep groundwater samples along the flow path. The ORP decreases progressively from the piedmont plain, through the central plain, to the littoral plain (Figure 3).

Total dissolved manganese (Mn_T_) concentrations are for the most part higher in groundwater from the central and the littoral plains as compared to those from the piedmont plain (Figure 3b). For shallow groundwater, Mn_T_ concentrations range from <0.01 µmol/L to 1.44 µmol/L (average 0.12 µmol/L) in the piedmont plain, 0.02 µmol/L to 30.64 µmol/L (average 7.12 µmol/L) in the central plain, and 0.69 µmol/L to 0.93 µmol/L (average 0.81 µmol/L) in the littoral plain, showing a general increasing trend along the flow path (Figure 3b). Deep groundwater has Mn_T_ concentrations lower than 0.02 µmol/L in the piedmont plain with the exception of sample 16-20 (12.96 µmol/L). In comparison to those of shallow groundwater, deep groundwater Mn_T_ concentrations are lower in the central and littoral plains. In these areas, Mn_T_ ranges from 0.02 µmol/L to 1.47 µmol/L (average 0.37 µmol/L), and from 0.16 µmol/L to 0.42 µmol/L (average 0.26 µmol/L), respectively. Along the groundwater flow path, Mn_T_ concentrations decrease during the first 30 km, and then increase to their highest concentrations at a down-gradient of approximately 50 km, before a progressive decline for the remainder of the sampled path (Figure 3b).

Total dissolved iron (Fe_T_) concentrations are higher than Mn_T_ concentrations in the piedmont, but comparable in the central and littoral plains (Figure 3b). Shallow groundwater has Fe_T_ concentrations in the range of 0.18 µmol/L to 1.62 µmol/L with an average of 0.56 µmol/L in the piedmont plain. In the central and littoral plains, Fe_T_ concentrations range from 0.23 µmol/L to 212.21 µmol/L (average 17.29 µmol/L), and from 3.71 µmol/L to 4.96 µmol/L (average 4.33 µmol/L), respectively. Total Fe concentrations of deep groundwater range from 0.25 µmol/L to 0.75 µmol/L (average 0.44 µmol/L) in the piedmont, and from 0.38 µmol/L to 7.51 µmol/L (average 1.94 µmol/L) and from 1.31 µmol/L to 51.74 µmol/L (average 13.45 µmol/L) in the central and littoral plains, respectively. Trends of Fe_T_ concentrations are similar to those of MnT concentrations along the flow path. It must be emphasized that, although measurable REE sorption on hydrous Fe oxides (HFO) at low pH (i.e., pH < 4) has been shown in previous experimental studies [28,31,35], modeling calculation performed using a recently built model [58] indicates that generally <2% of groundwater REEs are able to be complexed by HFO (except for sample 16-30 and 16-43 in this study) (see Figure S1). Therefore, from the viewpoint of scavenging modeling, REE complexation on HFO colloids is not considered in the present study. This flow path study only focuses on HMO.

### 3.2. Groundwater REE Signatures

Rare earth element concentrations are shown in Tables S3 and S4, respectively. In this study, Nd (selected as a tracer) concentrations range from 16 pmol/L to 754 pmol/L in shallow groundwater, with an average of 131 pmol/L, and from 27 pmol/L to 1468 pmol/L in deep groundwater aquifers, with an average of 246 pmol/L. Along the path, the trend of Nd is quite similar to that of ∑REE concentrations (Figure 3c). Specifically, shallow groundwater Nd concentrations show a decrease during the first 70 km, and then an increase down-gradient before a decrease along the remaining path (Figure 3c). The trends for deep groundwater Nd concentrations differ from those of shallow groundwater. Following a slight increase during the first 30 km, the Nd concentration decreases gradually over a distance of approximately 110 km. For the remainder of the sampled transect, Nd concentrations show greater fluctuation (Figure 3c). Overall, higher Nd concentrations are observed in the central plain, as compared to those of the piedmont and littoral plains.

**Figure 4:**
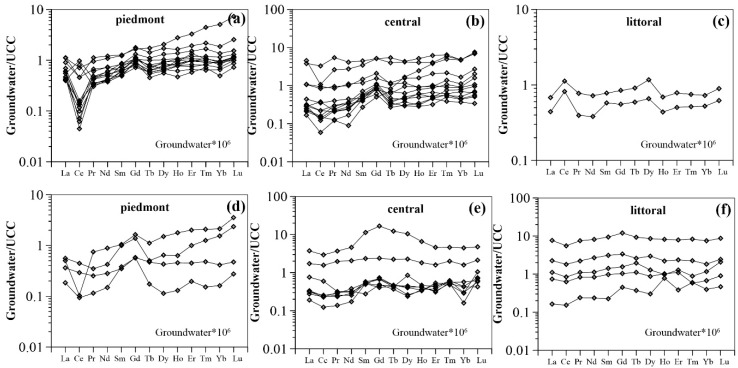
Upper continental crust (UCC)-normalized rare earth elements (REE) patterns for groundwater samples from three zones. ((**a**), (**b**), (**c**): shallow groundwater samples; (**d**), (**e**), (**f**): deep groundwater samples).

Rare earth element concentrations were normalized using the average REE composition of the upper continental crust (UCC) [60]. Fractionation indices [(Gd/Nd)_UCC_ and (Yb/Nd)_UCC_] were used as a measure of groundwater REE fractionation with respect to UCC. As presented in Figure 4, shallow groundwater REE patterns are characterized by middle REE (MREE) and heavy REE (HREE) enrichment in the piedmont plain, as indicated by (Gd/Nd)_UCC_ >1 and (Yb/Nd)_UCC_ >1 (i.e., values between 1.4 and 2.2 and between 1.2 and 4.2, with average values of 1.9 for both (Table S3)). Increased enrichment in HREE and MREE are observed in the central plain, with average (Gd/Nd)_UCC_ and (Yb/Nd)_UCC_ ratios of 2.8 and 3.4 respectively. A flat REE pattern is described for the littoral plain, being therefore much less enriched in HREE with respect to the piedmont and central groundwater samples (1.0 < (Yb/Nd)_UCC_ < 1.3). An overall MREE enrichment is characteristic of these samples as highlighted by the (Gd/Nd)_UCC_ ratios (1.2 < (Gd/Nd)_UCC_ < 1.5) (Figure 4c). For groundwater from deep aquifers, all REE patterns are consistently enriched in MREE, with (Gd/Nd)_UCC_ ratios of from 1.2 to 3.8 (Table S4). An overall decreasing trend in (Gd/Nd)_UCC_ ratios is observed as a function of distance from the recharge zone (Figure 3d). Significant enrichments in HREE are observed in the piedmont, where normalized ratios of (Yb/Nd)_UCC_ range between 1.1 and 3.6 with an average of 2.2. However, groundwater from the central and littoral plains exhibits relatively complex patterns of HREE enrichment, although (Yb/Nd)_UCC_ ratios are largely close to 1 (Figure 3d). Therefore, the UCC-normalized REE patterns for the investigated groundwater samples slope upward with increasing REE atomic number in the up-gradient regions of the flow path. However, they evolve into relatively smooth increases in normalized LREE concentrations with groundwater flow. This is better observed in shallow rather than in deep groundwater.

Another striking feature of groundwater REE patterns is that well-developed negative cerium anomalies (Ce/Ce* = [Ce]_UCC_/([La]_UCC_×[Pr]_UCC_)^0.5^) are commonly observed in the piedmont shallow groundwater, with the exception of two samples (i.e., 16-18 and 16-19). These samples have positive cerium anomalies with Ce/Ce* values of 2.3 and 2.0, respectively. Shallow groundwater from the central plain (average Ce/Ce* = 0.8) shows a smaller negative cerium anomaly compared to that of piedmont groundwater (average Ce/Ce* = 0.6), except for sample 16-22 (Ce/Ce* = 1.5) (Table S3). However, littoral shallow groundwater samples exhibit positive cerium anomalies (1.6 to 2.0). On the other hand, cerium anomalies of deep groundwater samples range between 0.2 and 1.2 with an average of 0.8 (Table S4). In the region proximal to the recharge area, where ORP values are highly variable, the cerium anomaly changes substantially. However, it stays relatively constant with groundwater flow from the recharge zone. There is only one exception (i.e., sample 16-39) which shows a positive cerium anomaly at a distance of 110 km from the recharge zone (Table S4).

### 3.3. Modeling Results

**Figure 5:**
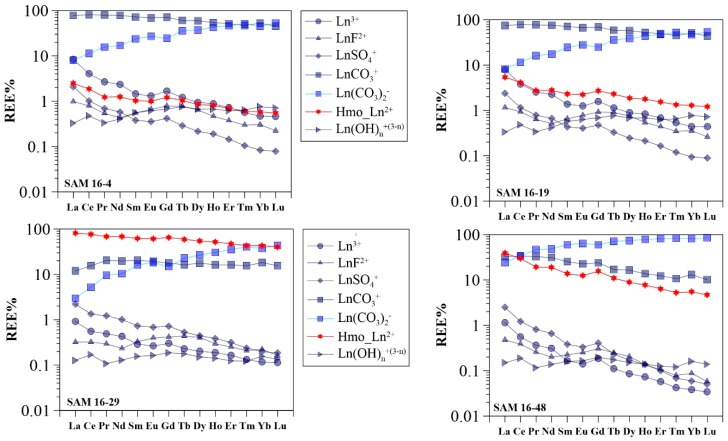
Results of speciation modeling of the REE in groundwater samples (Ln represents any of the REEs).

**Figure 6:**
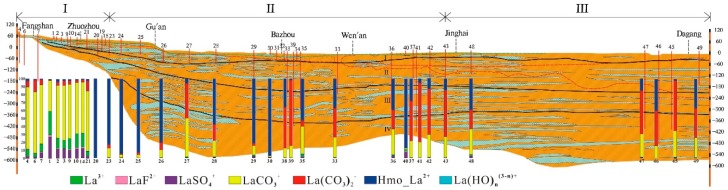
Speciation of groundwater La and its proportion of binding with hydrous manganese oxyhydroxides (HMO) as a function of distance from the charge area.

Results of speciation modeling involving REE surface complexation to HMO are presented in Figure 5. The model predicts that REE are predominantly complexed with carbonates at low dissolved Mn concentrations (< 0.1 µmol/L), whereas a significant amount of REE is complexed by HMO at higher Mn concentrations (≥0.1 µmol/L). Specifically, for the majority of the piedmont groundwater samples (i.e., those with low pH values and low Mn concentrations), carbonate complexes (LnCO_3_^+^ and Ln(CO_3_)_2_^−^) are the major REE species. The sulfate complex (LnSO_4_^+^) and the trivalent forms (Ln^3+^) are also suggested to be present at significant levels, especially for LREE. More specifically, average proportions of LaCO_3_^+^, La(CO_3_)_2_^−^, LaSO_4_^+^, and La^3+^ in these groundwater samples are modeled to 7%, 58%, 9%, and 13%, respectively, whereas LuCO_3_^+^, Lu(CO_3_)_2_^−^, LuSO_4_^+^ and Lu^3+^ species are present at levels of 41%, 44%, <1%, and <1%, respectively. Although HMO-complexes are universally insignificant with fractions mostly less than 5%, in groundwater with high Mn concentrations (i.e., samples 16-18 and 16-20), the bulk REE are predicted to be strongly complexed by Mn oxides (e.g., the total HMO-complexes of La, ranging from 74 to 100%; Figure 6). For groundwater samples from the central plain, the proportion of LnCO_3_^+^ decreases, while Ln(CO_3_)_2_^−^ fractions increase with increasing pH. Significant fractions of REE are observed associated with HMO through the speciation modeling. These results show that the sum of the HMO complexes accounts for 2% to 97% (average 69%), and 2% to 82% (average 31%) for La in shallow and deep groundwater samples, respectively (Figure 6). Surface complexation of REE with HMO persists in the down-gradient groundwater samples (i.e., from the littoral plain) (Figure 6) with an average proportion of La-HMO complexes of 38% in the shallow groundwater samples, and of 17% in the deep groundwater samples. In the latter samples, speciation is predicted to be dominated by carbonates. In all groundwater samples, the importance of HMO-complexes (i.e., Hmo_Ln^2+^) decreases with increasing atomic number across the REE series (e.g., the average Hmo_La^2+^ of 31% and average value of Hmo_Lu^2+^ of 15%). All the other potential inorganic complexes including LnF^2+^, LaSO_4_^+^, LnOH^2+^, and Ln(OH)_2_^+^ display consistently down-slope trends.

## 4. Discussion

### 4.1. Groundwater REE Mobility along the Flow Path

**Figure 7:**
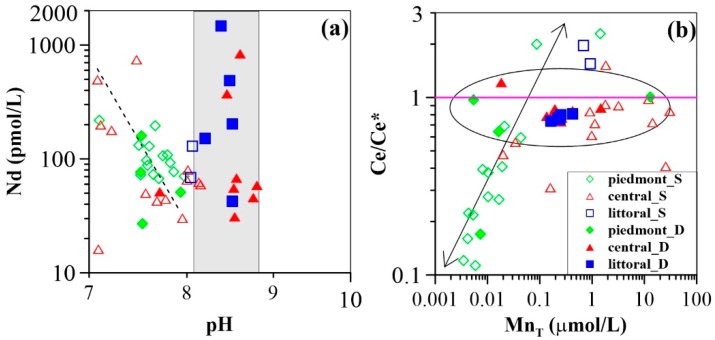
Concentrations of Nd as a function of pH (**a**); cerium anomaly (Ce/Ce*) as a function of Mn_T_ (**b**).

Groundwater REE concentrations change substantially with groundwater flow in both shallow and deep aquifers (Figure 3c). This effect appears to be correlated with changing pH. The strong pH dependence of REE concentrations has been observed in shallow and deep piedmont groundwater samples (except for sample 16-34) (Figure 7a). This shows that shallow groundwater REE concentrations decrease progressively from the recharge zone to a distance of approximate 70 km, and they increase as the pH increases with groundwater flow (Figure 3a,c). However, REE concentrations of deep groundwater samples from the central and littoral plains span over a wide range (i.e., 31 pmol/L ≤ Nd ≤ 1468 pmol/L) within a narrow range of pH (8.2 to 8.8) (Figure 7a). Consequently, the variations in groundwater REE concentrations is attributed to changes in pH.

Studies have showed that groundwater REE signatures might be acquired from the aquifer in recharge zones [21,25]. In these settings, acidic meteoric water, interacting with CO_2_ from the atmosphere, has a pH of ~5.7 after attaining equilibrium [61]. The uppermost soil water has a low pH in the range of 4 or 5 due to the decay of organic matter [62]. These acidic and oxidized fluids then give rise to chemical weathering and dissolution of minerals in aquifer rock/sediments. Therefore, REE as well as major and trace elements are mobilized from the host rock during acidic and oxidized water percolation. The investigated groundwater samples have a pH ranging between 7.1 and 8.8. Samples in and near the recharge zone generally have a lower pH than those from the down-gradient (Tables S1 and S2). Therefore, aquifer sediments would be the principal source of REE in groundwater via dissolution/weathering reactions of REE containing minerals. The most plausible mineral phases could be calcite/dolomite and aluminosilicate (i.e., feldspar) [52]. Geochemical modeling reveals that groundwater samples are all unsaturated with respect to amorphous silica (SiO_2(a)_) with saturation indices (SI) ranging from −1.3 to −0.5 and from −2.3 to −0.7 in shallow and deep groundwater samples, respectively. Calcite and dolomite appear to be saturated in groundwater. The only exception is sample 16-1, nearest to the recharge zone of the flow path (Figure 1). This groundwater was found to be unsaturated with respect to both carbonate minerals. Consequently, dissolutions of calcite and dolomite are favorable in the recharge zone and that silicate dissolution occurs along the entire flow path. Hence, groundwater REE might generate via dissolution of calcite and dolomite before the groundwater becomes over-saturated with respect to these minerals. This would occur within the first 34 km in shallow aquifers because beyond this distance, the groundwater can already be over-saturated with respect to calcite and dolomite. With groundwater flow (34 km) and infiltration in deep aquifers, dissolution of silicates may be the most important source of REE in these groundwater samples [25]. This may be the most plausible explanation for the substantial variation in REE concentrations for groundwater with pH values above 8.2 (Figure 7a).

Therefore, after being mobilized from solid phases in the recharge zone, REE preferentially form aqueous carbonate complexes (LnCO_3_^+^and Ln(CO_3_)_2_^−^) at higher pH conditions. The preferential stabilization of MREE and HREE as compared to LREE leads to MREE- and HREE-enrichment of groundwater. In the central plain (90 km to 130 km), however, it should be noted that enhanced complexation of the REE by LnCO_3_^+^ can occur in shallow groundwater. This would be a result of the lower pH values observed in these shallow groundwater samples as shown in Table S1. This further explains the reason for the greater enrichment of MREE and HREE in the central shallow groundwater, indicated by the (Gd/Nd)_UCC_ and (Yb/Nd)_UCC_ ratios. In contrast to groundwater flow in shallow aquifers and to that infiltrating through upper layer aquifers, deep groundwater shows an alkaline pH arising from chemical weathering of aluminosilicates, including feldspars. Under these conditions, deep groundwater REE speciation is dominated by negatively charged Ln(CO_3_)_2_^−^. This result is consistent with deep aquifer groundwater properties found in the central and littoral plains (50 km), as their recorded pH values (except for groundwater sample 16-29) are above 8.2 (Table S2). In consequence, the preferential mobilization of MREE and HREE over LREE in negatively charged carbonate forms (e.g., Ln(CO_3_)_2_^−^) contributes to the MREE and HREE enrichment in deep groundwater samples from the central and littoral plains [63]. Furthermore, with groundwater pH increase (i.e., alkaline conditions), the surface sites of Mn oxides/oxyhydroxides progressively become negatively charged, as the pH_PZC_ of Mn oxides (e.g., MnO_2_) is approximately 4.0 [36,64]. Similar cases have previously been reported previously [21,25]. These studies indicated that solution carbonate complexation (especially Ln(CO_3_)_2_^−^) of the REE was sufficient to overcome, to a certain extent, the binding affinity of the REE to mineral surface sites within aquifer sediments under alkaline conditions. This mechanism would thus favor conservative REE transport along the groundwater flow path. Consequently, changes in groundwater REE patterns along the flow path can be for the most part attributed to differences in solution carbonate complexation controlled exclusively by groundwater pH.

### 4.2. Controls on Groundwater REE Signatures: Roles of Hydrous Manganese Oxyhydroxides

**Figure 8:**
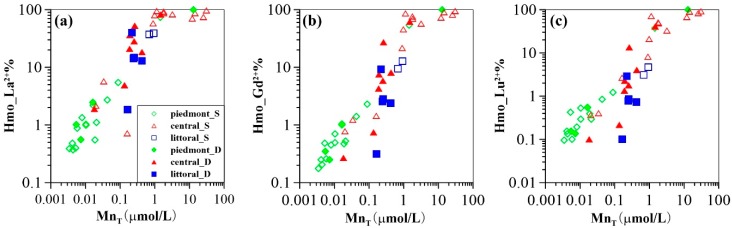
Proportion of La (**a**), Gd (**b**), and Lu (**c**) binding with HMO as a function of Mn_T_ concentrations.

The low pH_pzc_ (i.e., pH < 4.0) of Mn oxides (i.e., MnO_2_) [36,64] makes them one of the dominant scavengers for aqueous REE [65,66,67]. More importantly, Ce anomalies are positively correlated with Mn concentrations in groundwater (e.g., especially in shallow groundwater) as shown in Figure 7b. Accordingly, the proportions of the REE (i.e., La, Gd, and Lu) complexed to HMO are predicted to increase significantly with increasing groundwater Mn concentrations (Figure 8). These modeling results strongly suggest the role of HMO should be therefore quantified in order to further characterize the mechanisms of groundwater REE mobilization in this study.

A key feature of the modeling results is that the proportion of HMO complexes decreases downward as a function of REE atomic number. This property may be considered independent of the natural pH, Mn concentrations, and ionic strength, as comparable results were obtained from the modeling of different groundwater compositions from samples collected along a single flow path, even though groundwater chemistry changes substantially (Tables S1 and S2). Consequently, the effect of HMO on REE signatures can be interpreted from two perspectives: namely, (1) scavenging of REE by Mn oxyhydroxides in groundwater from the portion of the aquifer nearest to the recharge zone; and (2) REE complexed to Mn oxyhydroxides with groundwater flow and infiltration.

The REE were found to be principally mobilized in the recharge zone, where Mn oxyhydroxides formed particles/colloids in groundwater after being mobilized simultaneously. Manganese oxyhydroxides nucleate and form larger particles under oxidizing conditions in and nearest the recharge zone. These can readily precipitate or be transported with groundwater flow. From this study two main roles of HMO can be established with respect to their regulation of REE fractionation in oxidizing conditions nearest to the recharge region: (1) the preferential uptake of LREE relative to HREE and MREE by Mn oxyhydroxides during REE mobility; and (2) the preferential co-precipitation of LREE over HREE and MREE together with Mn oxyhydroxides. Both of these processes remove LREE from solution, and therefore generate HREE and MREE enrichment in groundwater, as in the piedmont area (Figure 4a,d). These REE removal mechanisms occur in conjunction with the low concentrations of dissolved Mn in these oxidizing groundwaters (Figure 3b). It is unclear, however, which of these processes exerts a more significant impact on REE fractionation. The low Mn concentrations (generally less than 0.1 µmol/L) and the oxidizing conditions suggest that co-precipitation of Mn oxyhydroxides is the common process occurring in the piedmont. On the other hand, the positively charged REE species (LnCO_3_^+^, LnSO_4_^+^, LnOH^2+^, and Ln^3+^), represent the main aqueous REE components in the piedmont groundwater samples (Figure 6), are predicted to account for more fractions of LREE than those of HREE and MREE (Figure 6 and Figure 7). Sorption of these positively charged aqueous REE species by negatively charged Mn oxides would further lead to enrichment of the HREE and MREE as compared to that of LREE in groundwater. The decreasing REE concentrations within the first 50 km of the flow path in the shallow aquifer (Figure 3c) is further evidence that the predominant REE sorption is by Mn oxyhydroxides.

**Figure 9:**
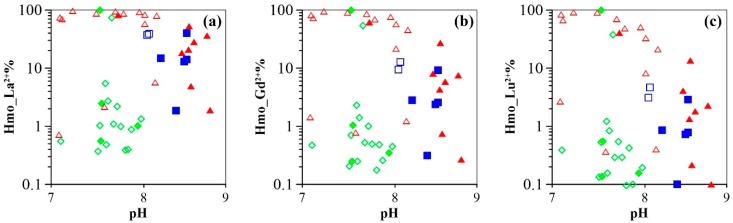
Proportion of La (**a**), Gd (**b**), and Lu (**c**) binding with HMO as a function of pH.

With groundwater migration (beyond 50 km) and infiltration (deep aquifers), the proportion of REE scavenged by Mn oxyhydroxides in groundwater increases with increasing Mn concentration, the reason being that the large size Mn oxyhydroxide particles, transported from up-gradient, changes their surface properties with the decrease in redox potential along the flow path and its lower value in deep aquifers (See ORP trends in Figure 3a,b) [24]. The particles favor scavenging of REE because of their elevated mobility and increased reactive surface area. Consequently, REE surface complexation with Mn oxyhydroxides particles competes with REE complexation with carbonate aqueous species. These processes would then be responsible for constraining groundwater REE concentrations and fractionation patterns to groundwater flow and infiltration (i.e., the central and littoral plains). The model reveals that larger proportions of REE are associated with HMO in groundwater samples collected from the down-gradient groundwater flow path (Figure 6). The remaining aqueous REE (Ln^3+^) form a dicarbonate complex (Ln(CO_3_)_2_^−^) at alkaline groundwater pH (e.g., pH > 8.0) (Figure 6). These results show that REE dicarbonate complexation coexists with HMO surface complexation in alkaline groundwaters. The proportion of REE binding to HMO shows typical patterns, smoothly declining downward across REE series. This implies preferential scavenging of LREE by Mn oxyhydroxides as compared to HREE and MREE, as shown by a previous experimental study [38]. Because the absolute concentrations of LREE are higher than those of MREE and HREE, competitive reaction between individual REE leads to HREE- and MREE-enrichment in solution, as expected for groundwater. However, REE patterns tend to be flat towards the end of the flow path, especially in deep aquifers of the latter two plains (Figure 4 and Figure 3d). This could be attributed to REE complexation competition with carbonates. Indeed, the proportion of dicarbonate complexes increases upward to a greater extent for the LREE as compared to that of the MREE and HREE with increasing REE atomic number (Figure 5). These two contrasting patterns suggest that REE solution complexation by carbonate suppresses REE surface complexation by Mn-oxyhydroxides. The strong competition between solution and surface complexation is primarily driven by solution pH. This is clearly evidenced by a decrease in the proportions of HMO complexes (Hmo_Ln^2+^) (Figure 9) and the increasing trend for REE carbonate complexes (Ln(CO_3_)_2_^−^) (Figure S2), both as a function of pH. This is best observed when pH rises above 8.0, as it is for groundwater from the central and littoral deep aquifers. The competitive effect between carbonate solution complexation and Mn-oxide surface complexation has been previously revealed by an experimental study that indicated that the rate of REE binding to δ-MnO_2_ was slower with respect to the carbonate-free systems [39]. Therefore, the weaker enrichment of HREE and MREE observed near the discharge zone can be attributed to a decreased complexation of the REE by HMO, which results from the competition for the REE, generated by the presence of carbonate ligands. This is consistent with the REE speciation modeling results that highlight a predominance of dicarbonate species for sampling locations near the end of the flow path (Figure 6).

### 4.3. Influence of Hydrous Manganese Oxide Content

**Figure 10:**
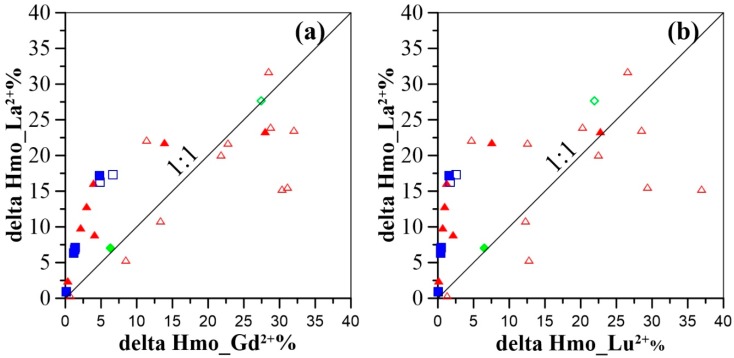
Delta fractions of Hmo_La^2+^ as a function of delta fractions of Hmo_Gd^2+^ (a), and Hmo_Lu^2+^ (b) (delta REE-HMO fractions were obtained by decreasing 50% of the dissolved Mn invoked in the model (Ln% sorbed _Total Mn_—Ln% sorbed _50% Mn_). It should be noted that those groundwater samples with Mn concentrations less than 0.1 μmol/L were not considered).

Apart from the strong influence of pH, HMO concentration is another important factor influencing REE complexation behavior. During the modeling calculation performed in this study, HMO was precipitated from measured Mn in groundwater samples with an equilibrium constant log K value of 41.38 [68]. The investigated flow path has higher concentrations of Mn in the central and littoral plains as compared to those in the piedmont areas (See Tables S1 and S2). The stronger affinity of suspended colloidal or particulate HMO for REE, revealed by the modeling calculations, facilitates REE mobility in groundwater along the flow path. This may account for the substantial changes in REE concentrations observed in this study (Figure 3c,d). To elucidate the influence of HMO concentrations on REE fractionation, modeling results of 50% Mn (samples with Mn concentrations greater than 0.1 µmol/L) being precipitated as HMO was compared to those of total Mn as HMO. The comparative results suggest that the proportion of the REE binding to HMO (Hmo_Ln^2+^) decreases more for the LREE as compared to that of the MREE and HREE when Mn was decreased by 50% (Figure S3). To be more specific, take shallow and deep groundwater samples (labelled 16-28 and 16-42, respectively) for example. These samples have pH values of 8.0 and 8.5, and Mn concentrations of 0.90 µmol/L and 0.42 µmol/, respectively. Considering 50% Mn as HMO in the model, Hmo_La^2+^, Hmo_Gd^2+^ and Hmo_Lu^2+^ proportions decrease by 22%, 10%, and 5% for the groundwater sample 16-28, and by 6%, 1%, and <1% for samples 16-42, respectively (Figure 10). These results imply that higher concentrations of LREE would be scavenged compared to those of MREE and HREE with increasing levels of HMO. The majority of the groundwater samples from the central and littoral plains show increasing amounts of HMO for REE scavenging. Piedmont groundwater shows REE co-precipitation with Mn oxyhydroxides. This suggests decreasing levels of HMO, as discussed previously [25,44]. It should be noted, however, that a small proportion of central shallow groundwater samples show no significantly incongruent declines for Hmo_Ln^2+^% (Figure 10), when we consider the hypothesis that only 50% of the total Mn is expressed as HMO. This is attributed to the high contents of HMO playing a crucial role in the scavenging of the whole groundwater REE, in spite of a constraint by the low pH condition (See Table S1). Although groundwater normalized REE patterns are observed to be fractionated to a lesser extent with groundwater flow and infiltration (discussed above), absolute concentrations of REE as well as formation of carbonate, hydroxide, or even organics complexation with HMO (not considered in the model) may be a constraint in reality. Nevertheless, these modeling results are generally consistent with those obtained by Decrée et al. [48], indicating a more significant fraction of LREE (i.e., La) complexed to HMO in the higher HMO content case (i.e., 10^−4^ mol/L compared to 10^−5^ mol/L). The influence of HMO content on metal speciation was studied through a modeling approach with the assumption (proposed by Cancès et al. [69]) that 50% of the measured Mn is considered HMO in the model [70]. More recently, the roles of various colloids including HMO particles in transport of trace elements and REEs in coastal groundwater were investigated thoroughly by Kim and Kim [71]. A comprehensive evaluation of REE-HMO proportions would be helpful to better understand REE aqueous speciation in natural groundwater.

### 4.4. Model Limitations

The proposed model published by Pourret and Davranche [36] based on the pioneering work of Tonkin et al. [72] has limitations. Indeed, HMO does not reflect exactly the occurrence of manganese oxide in nature: the sorption properties may be different depending on the nature of oxide (e.g., types and densities of functional groups; see discussion in Tonkin et al. [72]). Apart from these intrinsic known limitations of the HMO model, the redox properties of Ce(III) and the influence of organic matter (generally <1 mg/L) were not taken into account for REE speciation calculation in this study. This explains why no Ce decoupling from La and Pr during HMO complexation was observed (Figure 5). However, a significant accumulation of Ce on the surface of Mn oxide-containing particles/colloids (i.e., ferromanganese nodules) is attributed to its preferential removal from solution by HMO, with respect to La and Pr [63]. In sorption experiments with Mn oxyhydroxides, a negative Ce anomaly was observed in solution and a positive Ce anomaly was observed for solid phases, due to the oxidative scavenging of Ce(III), and as a less soluble form, Ce(IV) [27,38]. However, in the presence of organic matter (e.g., humic acids), oxidation scavenging of Ce(III) by Mn oxides does not usually occur [66,73,74]. As such, in the presence of organic matter, negative Ce anomalies in solution, but no Ce anomalies in solid phases, were observed [45]. Recently, the roles of microorganisms in Ce(III) oxidation upon Ce sorption onto Mn oxides were also studied in the presence of organic-ligand-producing bacteria [75] and *Pseudomonas fluorescens* cells [76]. The results of these studies demonstrate that the oxidized Ce(IV) by Mn oxide was bound to organics being stabilized in solution rather than sequestrated in Mn oxide surfaces in the presence of microbial activity [77]. The results of the modeling of Ce speciation in solutions suggest that geochemical models may be useful predictive tools for predicting Ce sorption and redox speciation in the presence of Mn oxide surfaces. Unfortunately, to date, no model can account for the development of the Ce anomaly.

The majority of the investigated groundwater samples show negative Ce anomalies (Tables S3 and S4), especially in the piedmont plain. These results contrast with those of REE inorganic speciation modeling. This further indicates that the organic substances may interfere with Ce sorption and redox speciation in the presence of Mn oxides. This effect may be accounted for by the inhibition of Ce redox reactions by the shielding of the Mn oxide reactive surfaces by an organic substance coating [42,66]. A similar shielding mechanism was recently reported for dissolved siderophores, showing no development of a positive Ce on the Mn oxide surfaces in the presence of siderophores [78]. In groundwater with low organic matter concentrations, no convincing evidence was found to suggest shielding on the Mn oxide surface. Therefore, no effects of organic matter on Ce(III) oxidation were observed. This is consistent with the modeling results obtained by Tang and Johannesson [79], which indicated that REE complexation with organic matter was insignificant in low-organic-carbon groundwater. However, a positive Ce anomaly was recorded for the last two groundwater samples (samples 16-48 and 16-49; Ce/Ce* of 1.6 and 2.0, respectively) (Table S3). This result can be attributed to the preferential complexation of Ce(IV) by dissolved carbonate ligands to the organic effect [74]. Indeed, carbonate preferential complexing with Ce(IV) over other potential ligands including organic ligands leads to positive Ce anomalies in some organic-poor alkaline waters [74]. Therefore, although Ce redox speciation on the surface of HMO and the effect of organics on Ce sorption onto HMO were not considered, the modeling results are in good agreement with previously reported experimental results [27,36,38], showing preferential scavenging of LREE relative to HREE and MREE. Prediction of Ce sorption and redox speciation at the surface of HMO requires further knowledge of redox equilibria and quantification of the stability of Mn oxide surface species. Aqueous chemistry of Ce(IV) species has recently been investigated using actinide analogues [80], which may be helpful for further prediction of Ce anomalies in the presence of Fe and Mn oxide surfaces.

## 5. Conclusions

Surface complexation modeling with high- and low-affinity sites was performed to describe REE sorption onto HMO. This was done using physico-chemical parameters from samples collected along a groundwater flow path of the North China Plain. The modeling results suggest that sorbed REE generally increases along the flow path from the piedmont to the littoral area. This result was obtained for a wide range of dissolved Mn concentrations (e.g., from < 0.01 µmol/L to 30.64 µmol/L), considered as HMO in the model. The role of HMO in controlling groundwater REE concentrations and fractionation patterns is addressed using surface complexation modeling. The preferential uptake of LREE over MREE and HREE by HMO led to the MREE- and HREE-enrichment of the investigated groundwaters. Proportions of REE complexed onto HMO show a more pronounced decrease for LREE as compared to MREE and HREE when only 50% of the total Mn concentration is considered HMO. REE sorption behavior on Mn oxide surfaces and REE solution speciation are controlled by groundwater pH. This parameter therefore regulates the fate and transport of REE along the studied flow path. A negative correlation is observed between total REE concentrations and pH in groundwater samples from the recharge zone (~50 km) and shallow aquifers. This is consistent with the dissolution of aquifer sediments, as in the recharge area, being the principal pathway generating REEs to groundwater. Modeled saturation indices indicate that groundwater initially acquired REE signatures from the dissolution of calcite and dolomite in the recharge zone before attaining equilibrium with respect to these carbonate mineral phases. Scavenging of LREE over MREE and HREE by Mn oxides, and preferential Mn oxyhydroxide co-precipitation of LREE over HREE and MREE, result in MREE- and HREE-enrichment of oxidizing groundwaters. Groundwater pH increases to alkaline pH values (pH > 8.0) due to chemical weathering of aluminosilicates, upon down-gradient flow and infiltration into deep aquifers. Groundwater REE concentrations span a wide range (e.g., 31 pmol/L ≤ Nd ≤ 1468 pmol/L) within a narrow pH range (8.2 to 8.8). The highly variable REE concentrations, for the most part, are attributed to REE complexation to dicarbonate species (Ln(CO_3_)_2_^−^). REE carbonate complexation is shown to suppress REE scavenging by Mn-oxyhydroxides. Thus, this reduces the amount of REE (i.e., LREE) scavenged by HMO. The results further show a progressive decrease in the fraction of REE complexed by HMO with increasing pH. In contrast, a steady increase in dicarbonate complexation is shown within the same pH range, suggesting competition between the HMO surface and carbonate species for REE complexation. These mechanisms reflect the main control of REE concentrations and fractionation patterns in deep aquifers in the central and littoral plains.

## Supplementary Materials

The following are available online at https://www.mdpi.com/1660-4601/16/13/2263/s1, Figure S1: Comparison of complexed REE by HMO and HFO ((a): La; (b): Gd; (c): Lu) (Modeling of REE surface complexation to HFO was performed using a recently built model (Liu et al., 2017. Appl. Geochem.)), Figure S2: Proportion of La(CO_3_)_2_^-^ (a), Gd(CO_3_)_2_^-^ (b), and Lu(CO_3_)_2_^-^ (c) as a function of pH, Figure S3: Patterns for Hmo_Ln^2+^ proportions modeled with total and 50% of the measured Mn (Ln represents any of the REEs), Table S1: Physiochemical parameters (including pH, Eh, EC, major ions and total dissolved solid (TDS)) of shallow groundwater samples (F, Cl, SO4, K, Na, Ca, Mg: mmol/L; FeT and MnT: µmol/L), Table S2: Physiochemical parameters (including pH, Eh, EC, major ions and total dissolved solid (TDS)) of deep groundwater samples (F, Cl, SO_4_, K, Na, Ca, Mg: mmol/L; Fe_T_ and Mn_T_: µmol/L), Table S3: REE concentrations (pmol/L) and fractionation parameters (Ce/Ce*, (Gd/Nd)_UCC_, (Yb/Nd)_UCC_ ) of shallow groundwater samples, Table S4: REE concentrations (pmol/L) and fractionation parameters (Ce/Ce*, (Gd/Nd)_UCC_, (Yb/Nd)_UCC_) of deep groundwater samples.
