# Supplementary material for: Role of Manganese Oxyhydroxides in the Transport of Rare Earth Elements Along a Groundwater Flow Path
Source: Int J Environ Res Public Health. 2019 Jun 26;16(13):2263. doi: 10.3390/ijerph16132263 (PMC6651366; doi:10.3390/ijerph16132263)
Supplement: Supplementary file 1 [file ijerph-16-02263-s001.pdf]

International Journal of
Environmental Research and Public Health

MDPI

Table S1. Physiochemical parameters (including pH, Eh, EC, major ions and total dissolved solid (TDS)) of shallow groundwater samples (F, Cl, SO_4, K, Na, Ca, Mg: mmol/L; Fe_T and Mn_T: μmol/L).

Zone	Sample No.	Distance (km)	Depth (m)	Aquifer group	ORP (mV)	pH	Conductivity (μs/cm)	F	Cl	SO_4	HCO_3	K	Na	Ca	Mg	Fe_T	Mn_T
Piedmont	16-1	33.1	4.5	A1	116.3	7.1	789	0.03	0.85	0.65	5.16	0.04	0.48	2.59	1.32	0.985	0.019
	16-2	33.9	12	A1	141.5	7.6	800	0.03	0.82	0.59	5.24	0.04	0.48	2.54	1.32	0.781	0.043
	16-3	34.7	40	A1	124.5	7.7	882	0.01	0.99	0.79	5.92	0.04	0.65	2.82	1.48	0.865	0.021
	16-9	36.8	50-60	A1	130	7.7	800	0.03	0.85	0.55	4.56	0.04	0.39	2.12	1.40	0.543	0.010
	16-8	37.4	>40	A1	115	7.7	654	0.04	0.51	0.64	4.39	0.04	0.35	1.82	1.07	1.619	0.017
	16-10	37.66	50-60	A1	50.2	7.8	826	0.03	0.82	0.54	3.97	0.04	0.39	2.64	1.40	0.177	0.003
	16-12	37.68	40-50	A1	103.1	7.8	764	0.03	0.73	0.50	4.00	0.04	0.35	2.64	1.32	0.231	0.005
	16-11	38.2	45	A1	90	7.9	827	0.03	0.82	0.54	7.56	0.05	0.39	2.59	1.36	0.186	0.006
	16-15	40.3	60-70	A1	110.3	7.6	671	0.03	0.65	0.42	4.44	0.04	0.43	2.17	1.11	0.235	0.004
	16-14	40.2	60-70	A1	129.2	7.5	728	0.03	0.85	0.45	4.72	0.04	0.48	2.32	1.15	0.186	0.004
	16-16	40.9	>40	A1	98.3	8.0	638	0.04	0.48	0.47	5.11	0.03	0.43	2.07	1.07	0.414	0.008
	16-17	44	>40	A1	63.4	7.5	1022	0.07	3.81	0.30	5.36	0.03	0.61	3.29	1.48	0.594	0.010
	16-19	47.6	70-80	A1	82.9	7.6	705	0.05	0.54	0.16	5.00	0.01	0.61	1.57	1.11	0.571	0.088
	16-18	48.3	40	A1	73.7	7.6	585	0.06	0.71	0.16	4.64	0.01	0.70	1.72	1.03	0.423	1.437
Central	16-21	51.9	90	A1	23.5	7.6	845	0.04	0.93	0.28	7.72	0.02	1.22	2.15	1.77	0.550	0.020
	16-22	56.5	75	A1	33.2	7.8	688	0.05	1.07	0.40	6.44	0.02	1.74	1.70	1.69	0.476	1.818
	16-23	62.3	100	A2	25.1	8.0	549	0.05	0.23	0.19	5.24	0.02	1.44	1.12	1.03	0.976	1.126
	16-24	67	80-90	A1	54.5	7.7	781	0.04	0.39	0.27	7.52	0.01	1.35	1.92	1.69	0.607	3.199
	16-25	70.5	90	A1	23.3	8.0	610	0.06	0.37	0.15	7.28	0.02	2.96	1.10	0.95	0.460	1.791
	16-26	75.8	53	A1	5.1	8.2	956	0.10	0.14	1.43	7.03	0.02	6.57	0.67	0.99	1.608	0.980
	16-27	78.7	80	A1	47.8	8.1	807	0.09	0.54	0.41	6.75	0.01	5.70	0.50	0.78	0.233	0.034
	16-28	82.8	70-80	A1	32.2	8.0	1652	0.13	1.72	2.80	10.60	0.02	14.57	0.70	2.10	0.646	0.899
	16-30	101.8	8.5-9	A1	-84.6	7.2	3040	0.09	8.15	4.05	15.16	0.15	10.09	6.69	3.08	212.205	30.635
	16-31	109.4	40	A1	40.1	7.1	5096	0.33	20.36	18.97	6.92	0.03	30.10	6.71	10.24	1.135	0.161
	16-32	113.4	40-50	A1	26.3	7.1	4609	0.17	9.76	15.24	8.47	0.08	23.79	4.39	8.56	2.946	12.012
	16-34	117.3	40-50	A1	35.5	7.1	8697	0.30	27.76	30.08	11.19	0.07	42.32	8.31	19.62	0.360	25.465
	16-40	132.1	10	A1	31.5	7.5	4308	0.37	17.46	7.32	12.39	0.08	20.70	4.54	7.16	2.546	14.434
Littoral	16-48	155.3	125	A1	-33.1	8.1	2138	0.27	15.94	4.15	7.18	0.03	24.84	0.80	1.19	4.960	0.925
	16-49	188	160	A1	-56.2	8.0	1058	0.32	1.52	0.88	11.05	0.02	19.57	0.22	0.21	3.707	0.685

International Journal of *Environmental Research and Public Health*

MDPI

Table S2. Physiochemical parameters (including pH, Eh, EC, major ions and total dissolved solid (TDS)) of deep groundwater samples (F, Cl, SO_4, K, Na, Ca, Mg: mmol/L; Fe_T and Mn_T: μmol/L).

Zone	Sample No.	Distance (km)	Depth (m)	Aquifer group	ORP (mV)	pH	Conductivity (μs/cm)	F	Cl	NO_3	SO_4	HCO_3	K	Na	Ca	Mg	Fe_T	Mn_T
Piedmont	16-4	7.3	400	A4	156.2	7.5	444	0.04	0.10	0.12	0.12	4.61	0.02	0.16	1.26	1.07	0.333	0.016
	16-6	15.9	350	A4	125.7	7.9	448	0.02	0.13	0.22	0.19	3.36	0.02	0.16	1.21	1.09	0.252	0.005
	16-7	27.5	230	A3	120.8	7.5	585	0.04	0.51	0.53	0.42	3.72	0.00	0.43	1.37	1.05	0.426	0.007
	16-20	51.0	100	A2	40.3	7.5	1926	0.04	10.63	0.09	0.60	7.39	0.04	1.61	5.51	2.80	0.745	12.960
Central	16-29	93.3	200	A3	-33.7	7.7	1709	0.18	4.91	0.03	2.77	6.80	0.02	10.79	1.06	1.81	2.306	1.473
	16-38	111.2	200	A3	-10.3	8.8	793	0.19	0.87	0.01	0.28	5.47	0.02	6.57	0.17	0.13	0.815	0.193
	16-39	110.7	370	A4	29	8.8	757	0.14	0.93	0.00	0.26	5.67	0.02	6.92	0.09	0.06	0.381	0.018
	16-33	114.0	200	A3	-19.5	8.5	734	0.21	1.95	0.02	0.57	2.61	0.02	5.26	0.20	0.17	1.787	0.428
	16-35	121.1	300	A3	-6.7	8.5	983	0.12	4.80	0.05	0.48	0.52	0.02	6.74	0.25	0.14	0.532	0.262
	16-36	127.6	300	A3	47.3	8.5	1367	0.16	8.32	0.00	0.24	4.28	0.03	11.79	0.20	0.12	1.348	0.135
	16-37	135.2	300	A3	-9.9	8.6	1622	0.17	9.48	0.03	0.31	4.83	0.04	12.88	0.28	0.22	0.806	0.189
	16-41	136.3	400	A4	-62.7	8.5	1503	0.25	6.04	0.03	0.25	7.92	0.03	12.96	0.20	0.35	7.513	0.251
Littoral	16-42	143.5	300	A3	-8.2	8.5	2099	0.30	13.06	0.04	0.51	4.52	0.04	16.83	0.28	0.16	8.187	0.422
	16-43	146.6	200	A3	-4	8.5	1812	0.26	10.30	0.04	0.43	5.67	0.02	14.44	0.35	0.59	51.740	0.244
	16-46	182.3	400	A4	-61.6	8.5	1592	0.07	5.56	0.03	0.48	5.72	0.04	11.61	0.21	0.18	2.607	0.222
	16-47	183.2	500	A4	-136.3	8.2	2358	0.22	8.41	0.08	2.43	6.64	0.07	18.66	0.22	0.07	3.424	0.253
	16-45	183.3	400	A4	-63.6	8.4	2114	0.49	7.84	0.09	2.53	5.33	0.04	16.66	0.25	0.20	1.305	0.164

International Journal of
Environmental Research and Public Health

MDPI

Table S3. REE concentrations (pmol/L) and fractionation parameters (Ce/Ce*, $(Gd/Nd)_{UCC}$, $(Yb/Nd)_{UCC}$) of shallow groundwater samples.

Zone	Sample No.	La	Ce	Pr	Nd	Sm	Gd	Tb	Dy	Ho	Er	Tm	Yb	Lu	ΣREE	Ce/Ce*	$(Gd/Nd)_{UCC}$	$(Yb/Nd)_{UCC}$
Piedmont	16-1	241.96	209.04	56.77	217.97	38.04	40.00	6.98	44.12	13.40	45.26	8.64	64.72	13.32	147.4	0.41	1.37	4.21
	16-2	194.95	210.47	33.43	129.71	24.94	31.86	4.47	28.18	6.67	20.93	3.26	17.39	2.80	102.69	0.60	1.83	1.90
	16-3	239.80	322.66	47.62	195.58	36.71	42.99	5.73	36.74	7.94	26.37	4.20	23.64	4.69	143.94	0.69	1.64	1.71
	16-9	149.45	72.58	24.27	106.49	26.00	25.44	3.96	22.09	5.21	16.26	2.31	14.56	2.34	68.74	0.28	1.78	1.94
	16-8	91.86	45.46	16.61	67.11	15.16	17.36	2.33	14.34	3.76	13.63	1.95	10.40	2.00	44.24	0.27	1.93	2.20
	16-10	128.58	28.19	22.00	108.85	21.95	26.77	2.64	20.12	4.61	18.59	2.07	12.02	2.00	58.46	0.12	1.83	1.57
	16-12	131.24	53.38	24.06	92.48	19.75	24.55	3.21	19.63	5.15	14.71	2.07	12.08	2.11	59.09	0.22	1.98	1.85
	16-11	100.21	20.41	17.03	77.09	24.94	21.11	2.52	16.92	4.12	13.21	2.13	11.33	2.17	46.11	0.11	2.04	2.09
	16-15	101.29	32.62	21.29	87.49	14.57	22.96	2.20	13.17	3.76	10.22	1.60	12.19	1.83	47.58	0.16	1.95	1.98
	16-14	129.58	61.45	30.45	131.93	18.36	25.88	3.15	14.83	4.79	13.09	1.78	11.38	1.83	65.24	0.22	1.46	1.22
	16-16	86.68	67.44	17.74	70.58	18.62	19.01	2.33	14.03	2.97	9.09	1.24	8.21	1.60	46.53	0.39	2.01	1.65
	16-17	89.05	65.87	17.81	72.59	17.56	21.49	2.52	15.57	4.18	13.75	1.78	11.56	2.06	49.18	0.38	2.21	2.26
	16-19	119.07	443.35	21.65	96.99	18.29	27.54	3.02	18.46	3.76	11.30	1.54	9.13	1.83	110.93	2.00	2.12	1.33
	16-18	85.74	364.13	15.33	73.00	15.56	21.49	1.82	12.00	2.30	7.95	1.42	6.30	1.31	86.85	2.29	2.20	1.22
Central	16-21	63.93	55.45	11.00	50.26	13.30	24.04	1.64	9.85	2.36	7.23	1.07	6.53	1.26	36.22	0.48	3.57	1.84
	16-22	61.19	171.15	10.79	44.30	16.69	20.73	1.38	6.34	1.39	4.36	1.01	5.61	0.91	49.67	1.52	3.50	1.79
	16-23	62.85	100.49	10.36	42.78	15.63	20.10	1.26	8.06	1.52	6.28	0.77	4.62	0.63	39.8	0.90	3.50	1.54
	16-24	69.18	101.06	15.05	65.45	21.15	26.65	1.89	9.29	2.97	9.03	1.18	8.78	1.20	48.44	0.71	3.04	1.90
	16-25	44.92	67.80	6.39	30.30	13.04	14.56	1.07	6.52	1.70	6.28	1.12	5.72	1.03	29.18	0.91	3.58	2.68
	16-26	49.24	72.51	14.83	59.76	11.84	20.22	1.45	10.83	2.91	6.88	1.48	9.94	1.77	38.57	0.61	2.52	2.36
	16-27	54.07	62.88	12.14	62.19	17.16	24.67	3.33	36.18	11.94	52.07	9.65	59.70	13.77	64.92	0.56	2.96	13.61
	16-28	95.10	159.94	20.16	80.28	14.10	18.95	2.52	20.31	4.49	13.09	2.72	14.10	3.66	65.31	0.83	1.76	2.49
	16-30	229.44	383.68	47.83	178.52	44.16	50.62	4.40	25.17	4.37	13.81	1.78	11.38	1.94	143.15	0.84	2.11	0.91
	16-31	982.68	487.24	131.22	493.41	103.22	123.50	21.65	93.17	24.80	84.90	12.55	60.56	12.75	244.57	0.31	1.87	1.74
	16-32	232.82	429.08	43.08	198.70	35.18	37.52	4.78	33.54	7.70	28.10	4.20	21.09	4.92	155.78	0.98	1.41	1.51
	16-34	36.21	26.69	6.10	16.08	8.11	12.08	2.83	13.48	3.82	12.02	2.07	11.73	2.91	23.24	0.41	5.62	10.37
	16-40	825.02	1502.3	273.65	745.29	133.01	123.50	16.05	90.52	19.64	55.66	11.42	61.03	12.63	324.59	0.72	1.24	1.16
Littoral	16-48	146.86	513.86	39.03	129.65	23.28	20.35	3.65	25.23	3.33	10.76	1.48	9.25	1.66	132.47	1.55	1.17	1.01
	16-49	95.03	373.98	19.87	68.64	17.29	13.35	2.39	14.15	2.12	6.94	1.01	6.65	1.14	88.71	1.97	1.45	1.37

International Journal of
Environmental Research and Public Health

MDPI

Table S4. REE concentrations (pmol/L) and fractionation parameters (Ce/Ce*, $(Gd/Nd)_{UCC}$, $(Yb/Nd)_{UCC}$) of deep groundwater samples.

Zone	Sample No.	La	Ce	Pr	Nd	Sm	Gd	Tb	Dy	Ho	Er	Tm	Yb	Lu	ΣREE	Ce/Ce*	$(Gd/Nd)_{UCC}$	$(Yb/Nd)_{UCC}$
Piedmont	16-4	39.67	42.68	5.82	26.90	11.37	13.74	0.69	2.46	0.61	2.69	0.30	2.08	0.51	21.62	0.61	3.82	1.09
	16-6	77.89	133.68	12.77	50.89	10.44	13.99	1.89	9.17	2.18	6.22	0.95	5.26	0.86	46.87	0.97	2.05	1.47
	16-7	108.85	47.46	37.54	159.32	31.19	39.75	4.53	32.31	8.61	27.74	4.03	27.28	6.46	79.54	0.16	1.86	2.43
	16-20	121.38	203.90	17.67	76.47	29.93	33.58	2.01	13.91	3.09	13.75	2.49	19.59	4.29	78.68	0.96	3.27	3.63
Central	16-29	72.50	111.19	11.71	51.65	17.23	16.53	1.82	9.66	2.00	4.48	1.01	2.02	1.26	43.62	0.87	2.39	0.56
	16-38	59.82	105.13	12.92	45.69	15.83	11.07	1.89	8.92	2.06	4.24	1.07	7.28	1.14	40.01	0.86	1.80	2.26
	16-39	164.21	273.06	15.97	58.51	8.18	11.70	1.45	5.17	1.76	5.20	1.18	3.81	1.03	78.10	1.21	1.50	0.92
	16-33	375.36	713.70	99.64	371.05	69.57	57.11	9.00	49.17	8.85	22.06	3.91	20.23	3.89	258.55	0.84	1.15	0.77
	16-35	41.11	56.38	6.88	31.06	15.43	9.73	1.76	5.60	1.64	7.35	1.01	5.37	0.80	26.84	0.76	2.34	2.45
	16-36	68.10	115.41	14.34	68.08	15.90	16.09	1.70	18.15	2.30	5.86	1.18	5.49	1.37	48.36	0.84	1.76	1.15
	16-37	68.46	103.63	15.33	55.53	14.83	17.81	1.89	8.62	1.70	6.58	0.95	3.70	1.94	43.45	0.73	2.39	0.94
	16-41	804.14	1331.1	186.01	833.33	341.45	403.05	49.33	224.37	31.95	62.90	9.00	56.40	8.74	512.03	0.79	3.61	0.96
Littoral	16-42	486.52	825.04	112.27	485.23	92.44	80.76	10.51	63.75	10.79	32.40	4.38	23.29	4.46	204.77	0.81	1.24	0.68
	16-43	240.38	382.97	55.92	203.62	42.10	37.52	7.87	27.63	4.73	17.70	1.72	14.79	3.83	149.53	0.75	1.38	1.03
	16-46	35.35	69.73	12.14	42.36	6.72	10.94	1.51	6.46	3.76	5.26	1.18	5.09	0.86	29.25	0.76	1.93	1.70
	16-47	160.40	287.05	41.94	150.58	29.06	24.99	4.47	18.83	4.97	15.07	1.12	8.50	1.66	107.49	0.80	1.24	0.80
	16-45	1648.6	2542.2	377.13	1468.4	281.32	290.05	37.56	183.57	39.23	108.75	16.16	94.89	15.83	1020.4	0.74	1.47	0.92

International Journal of
Environmental Research and Public Health

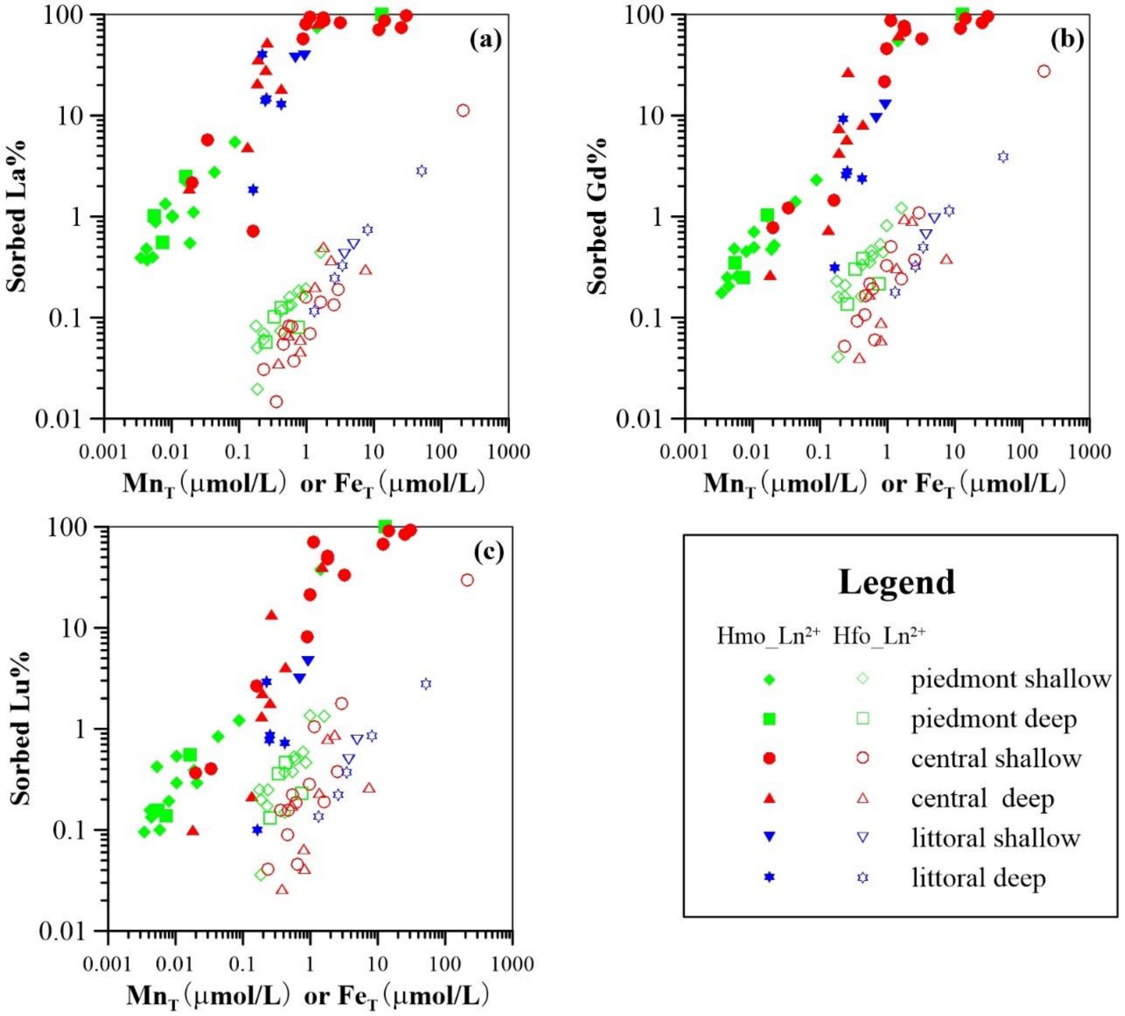

Figure S1. Comparison of complexed REE by HMO and HFO ((a): La; (b): Gd; (c):Lu) (Modeling of REE surface complexation to HFO was performed using a recently built model (Liu et al., 2017. Appl. Geochem.)).

International Journal of
*Environmental Research
and Public Health*

MDPI

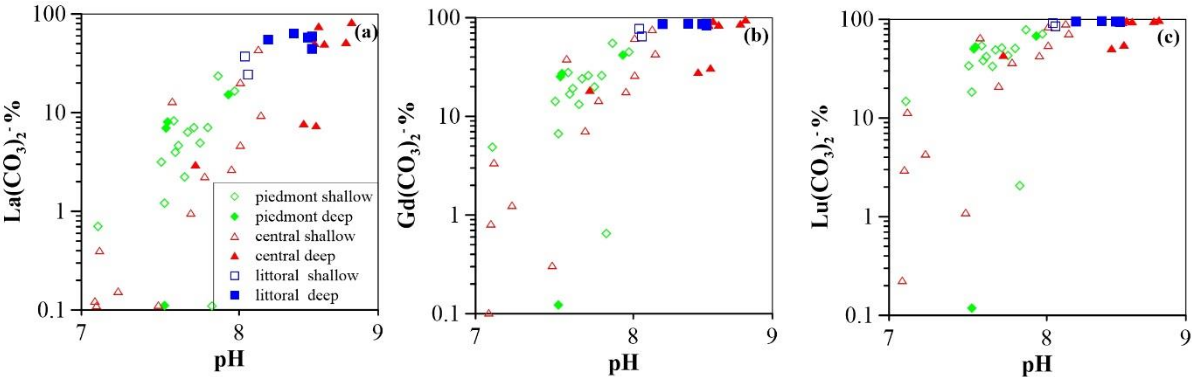

Figure S2. Proportion of $La(CO_3)_2^-$ (a), $Gd(CO_3)_2^-$ (b), and $Lu(CO_3)_2^-$ (c) as a function of pH.

International Journal of
Environmental Research and Public Health

MDPI

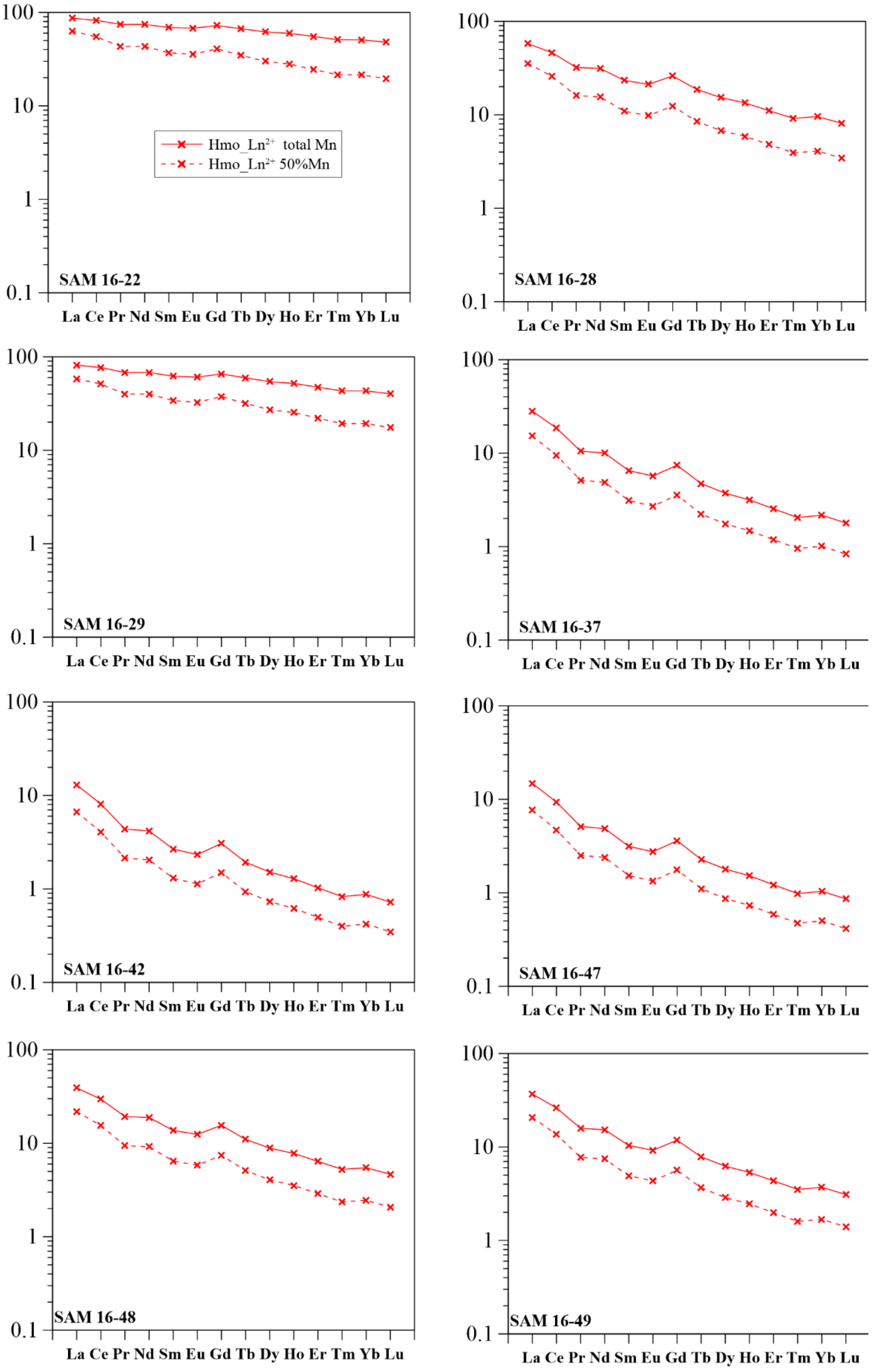

Figure S3. Patterns for Hmo_Ln^{2+} proportions modeled with total and 50% of the measured Mn (Ln represents any of the REEs).
